# A switch from α5β1 to αvβ3 integrin activity contributes to the development of a profibrotic mesenchymal phenotype in trabecular meshwork cells

**DOI:** 10.3389/fcell.2025.1730542

**Published:** 2026-01-30

**Authors:** Kassidy L. Johns, Jennifer A. Faralli, Mark S. Filla, Nandini Shah, Kate E. Keller, Donna M. Peters

**Affiliations:** 1 Department of Pathology & Laboratory Medicine, University of Wisconsin School of Medicine and Public Health, Madison, WI, United States; 2 Casey Eye Institute, Oregon Health & Science University, Portland, OR, United States; 3 Department of Ophthalmology & Visual Sciences, University of Wisconsin School of Medicine and Public Health, Madison, WI, United States

**Keywords:** aging, contractility, EndMT, integrins, trabecular meshwork

## Abstract

**Purpose:**

Fibrogenic changes in the trabecular meshwork (TM) are considered to be a major cause for the restriction in aqueous humor outflow from the anterior chamber associated with primary open angle glaucoma. In this study, we investigated whether integrin switching from α5β1 to αvβ3 integrin expression could initiate fibrotic-like changes in the TM that could restrict outflow.

**Methods:**

Human TM cells were isolated from young (<40 years) and old (>50 years) donor eyes. RT-PCR, western blots and immunofluorescence microscopy were used to evaluate levels of integrin and αSMA expression. Lentiviral shRNA vectors were used to knockdown α5 and β3 integrin levels. Paraffin embedded anterior segments of young and old donor eyes were used to evaluate αSMA levels *in situ*.

**Results:**

Studies revealed an age-related decrease in α5 integrin mRNA expression in TM cells. This loss was accompanied by an increase in αSMA mRNA and protein levels and an increase in activated αvβ3 integrin levels. Knockdown of β3 integrin mRNA and protein levels decreased the expression of αSMA mRNA and protein levels. Elevated mRNA levels of the EndMT biomarkers, *VIM*, *SNAI2*, and *TWIST1*, observed in older TM cells were decreased when β3 integrin was knockdown.

**Conclusion:**

These studies suggest that crosstalk between α5β1 and αvβ3 integrin signaling controls expression of αSMA mRNA and protein levels and that β3 integrins may play a role in the development of the fibrogenic phenotype in TM cells and associated with POAG.

## Introduction

Glaucoma is a family of optic neuropathies that result in irreversible optic nerve damage. It is the major cause of irreversible blindness worldwide. Nearly 76 million people have the disease, including >4 million in the United States (Tham et al., 2014; Ehrlich et al., 2024). In the United States, primary open-angle glaucoma (POAG) is the most common form of glaucoma. Like many other chronic age-related diseases, there is increasing evidence that fibrotic-like changes play a role in the pathogenesis of POAG. These changes are a consequence of cell-specific factors that result in unresolved inflammatory responses, uncontrolled remodeling of the extracellular matrix (ECM) and senescence or cell loss (Selman and Pardo, 2021; Filla et al., 2021). They are considered the primary cause for the elevation in intraocular pressure (IOP), a major risk factor in the development of POAG that if uncontrolled will damage the optic nerve leading to retinal ganglion cell death and ultimately causing blindness. In POAG, these fibrotic-like changes occur within the trabecular meshwork (TM), specifically within the juxtacanalicular tissue (JCT) and inner wall of Schlemm’s Canal (SC). The TM is a critical regulator of IOP as it acts as a pulsatile pump that controls the movement of aqueous humor (AH) out of the anterior chamber (Johnstone et al., 2021). Fibrotic-like changes during aging are thought to make the TM tissue stiffer (Last et al., 2011; Stamer et al., 2015) thereby causing the pulsatile properties of the TM to malfunction resulting in an elevation in IOP (Johnstone et al., 2021).

A major cause for these fibrogenic changes in the TM is thought to be elevated levels of transforming growth factor-β2 (TGFβ2) in aqueous humor. (Fuchshofer and Tamm, 2012; Li et al., 2022). Greater than half of POAG patients have elevated TGFβ2 levels in their aqueous humor (Tripathi et al., 1994; Picht et al., 2001; Tan et al., 2024). These changes are associated with a process called endothelial-to-mesenchymal transition (EndMT) that results in the development of a myofibroblastic phenotype (Islam et al., 2021; Yang et al., 2025). EndMT is characterized by the upregulated expression of specific cellular biomarkers, such as snail, twist, α-smooth muscle actin (αSMA), and vimentin, increased levels of ECM molecules, such as the EDA + isoform of fibronectin (EDA + FN) and collagen types I and IV, as well as the expression of the cytokines TGFβ and connective tissue growth factor (CTGF). Transition into this phenotype occurs in stages (Fang et al., 2021) and involves a constellation of signaling pathways including cell-matrix interactions with integrins (Machado-Costa et al., 2020).

Integrins are a family of receptors that sense changes in the mechanobiological environment of cells and act as critical modulators of processes that control outflow facility and IOP. These processes include the assembly and remodeling of the ECM (Singh et al., 2010; Sun et al., 2025), the contraction forces of the cellular actomyosin network (Tang and Anfinogenova, 2009; Roca-Cusachs et al., 2012) and the phagocytic properties of TM cells (Dupuy and Caron, 2008; Gagen et al., 2013; Peotter et al., 2016). To date, 14 different integrins have been identified in the TM tissue where they are localized throughout the TM, JCT and SC regions of the outflow pathway (Faralli et al., 2019b; van Zyl et al., 2020). The activity of integrins is tightly regulated by their conformation. An inactive integrin has a bent conformation while an active integrin is in an upright conformation and can engage ECM proteins, form focal adhesions, trigger actin polymerization and regulate several signal transduction pathways. Integrins rapidly fluctuate between active and inactive conformations in response to environmental conditions present in the TM such as pressure, stretch, and ECM composition, all of which can be altered in POAG (Stamer and Acott, 2012; Filla et al., 2017; Kechagia et al., 2019; Johnstone et al., 2021; Keller and Peters, 2022).

Numerous studies have shown that crosstalk between different integrins regulates their expression and activity (Diaz-Gonzalez et al., 1996; Kim et al., 2011; Faralli et al., 2022). Hence any change or loss in integrin expression, often referred to as integrin switching, is likely to affect the activity of existing integrins on the cell surface (Madamanchi et al., 2014) and, in some instances, contribute to an EndMT phenotype (Truong et al., 2014; Rapisarda et al., 2017). Recent studies have suggested that a switch in integrin expression and activity may be occurring in some aging TM cells and contributing to the development of a fibrogenic phenotype. These studies showed that an age-related loss of α5β1 integrin expression on the TM cell surface contributed to an increase in activity of the αvβ3 integrin (Johns et al., 2025). Cells expressing activated αvβ3 integrin were found to be more contractile and assembled αSMA into robust stress fibers. A comparison of αvβ3 integrin expression in glaucomatous TM cells and aged matched normal TM cells also showed more intense staining of αvβ3 integrin in focal adhesions in glaucomatous cells (Yang et al., 2025). These focal adhesions were larger and reminiscent of supermature focal adhesions observed in myofibroblasts associated with a fibrotic phenotype (Hinz, 2010; Hinz and Gabbiani, 2010). Finally, over expression of the active αvβ3 integrin increased the expression of the profibrotic genes associated with TGFβ2-induced glaucoma (Filla et al., 2017; Faralli et al., 2022; Faralli et al., 2023).

Among the integrins in the TM, the αvβ3 integrin is most likely to be involved in POAG (van Zyl et al., 2020; Filla et al., 2017; Zhou et al., 1999). The αvβ3 integrin is mostly concentrated in the inner wall of SC and the JCT, which is the primary site of resistant for aqueous humor outflow. The activity of αvβ3 integrin triggers many of the common changes associated with POAG (Filla et al., 2017; Peotter et al., 2016). The transition into a myofibroblast phenotype is often preceded by the activation of αvβ3 integrin (Hinz and Gabbiani, 2010; Hinz et al., 2019; Faralli et al., 2023; Goffin et al., 2006; Hinz, 2010). The knockdown of αvβ3 integrin decreased IOP in mice and activation of it increased IOP in both a porcine organ culture perfusion model and mice *in vivo* (Faralli et al., 2019a; Filla et al., 2021). Activation of αvβ3 integrin signaling also increases the deposition of EDA+FN into the ECM and the expression of TGFβ2 in TM cultures, both factors involved in POAG (Filla et al., 2019; Filla et al., 2021). It can also serve as a receptor for the growth factor CTGF, a downstream target of TGFβ2 in the TM that can upregulate IOP (Junglas et al., 2012; Junglas et al., 2009; Hennig et al., 2016). In addition, studies show that αvβ3 integrin is part of a secondary glucocorticoid (GC) response in GC-induced-glaucoma that regulates the formation of cross-linked actin networks (CLANs) and phagocytosis (Filla et al., 2009; Filla et al., 2006; Faralli et al., 2013; Gagen et al., 2013). Finally, studies have shown that its activity is associated with hic-5, a transcription factor involved in the TGFβ2-induced fibrogenic response in human TM cells (Pattabiraman and Rao, 2015).

In this study, we used human TM cells to investigate if switching integrin expression from α5β1 to αvβ3 impacts the development of a myofibroblast phenotype. Using shRNA lentiviral particles to knockdown expression of α5 or β3 integrins, these studies show that the expression of αSMA was dependent on both the level of α5 integrin mRNA and the activity of the αvβ3 integrin. When α5 integrin mRNA levels were high in young TM cells, we found that the expression of αSMA and the formation of αSMA stress fibers in TM cells was downregulated. In contrast, low levels of α5 integrin mRNA in TM cells triggered an increase in αSMA expression and stress fiber formation as well as an increase in the activated levels of αvβ3 integrin. In addition, there was an increase in the mRNA levels for EndMT biomarkers (*VIM*, *SNAI2*, and *TWIST1*). Conversely, low levels of β3 integrin mRNA in old TM cells caused a decrease in αSMA stress fibers and in the mRNA levels for *αSMA*, *VIM*, *SNAI2*, and *TWIST1.* Together, these studies suggest that integrin switching could be an early step in the development of the fibrotic-like phenotype associated with POAG.

## Materials and methods

### Cell culture

Human TM cells were isolated in accordance with the tenets of the Declaration of Helsinki from corneal rims or whole globes of cadaver eyes as previously described (Filla et al., 2006). Human tissue experiments complied with the guidelines of the ARVO Best Practices for Using Human Eye Tissue in Research (November 2021). Donor eyes and corneal rims were obtained from both the Lions Eye Bank of Wisconsin and VisionGift, Portland, OR. Tissues from both males and females were used and all donors were Caucasian with no known history of glaucoma or other ocular diseases. The sex, age, and cause of death of specific donors has been previously published (Johns et al., 2025). All the TM cell strains were judged to be TM cells based upon criteria previously described (Keller et al., 2018) and the upregulation of myocilin expression in response to dexamethasone was confirmed (Johns et al., 2025). Cells were grown in low glucose Dulbecco’s Modified Eagle’s Medium (DMEM Sigma-Aldrich) supplemented with 15% fetal bovine serum (FBS, Avantor-VWR), 2% L-glutamine (Sigma-Aldrich), 1% amphotericin B (Corning), 0.05% gentamycin (Sigma-Aldrich), and 1 ng/mL FGF-2 (Peprotech) and used between passages 5 and 8. Table 1 shows the cell strains used in this study and the percentage of cells in each strain expressing α5 integrin at the cell surface as determined by flow cytometry.

**TABLE 1:** Percentage of TM cells containing α5 integrin on cell surface. Donor nomenclature refers to nomenclature used in previous publication (Johns et al., 2025)

Cell strain	Donor	Gender	Age of donor	α5 integrin positive cells (%)
N17	N17RM.1	Male	17	91
N21	2021-0755	Female	21	96
N25	N25LM8.1	Male	25	98
N27	N27TM-6	Female	27	89
N27-2	N27TM-2	Female	27	94
N35	N35LM8.1	Male	35	98
N36	2017-0509	Male	36	93
N55	2018-1341	Male	55	99
N57	2021-1323	Male	57	92
N69	2020-0984	Male	69	96
N71	N71LF3	Female	71	88
N74	2021-1493	Female	74	40
N75	2021-1328	Male	75	98
N77	2022-0140	Female	77	51

### RNA isolation and RT-qPCR

Total RNA was isolated from TM cells using RNeasy Plus Mini Kit (Qiagen Inc, Germantown, MD), or TRIzol (Invitrogen), and reversed transcribed into cDNA using the High-capacity cDNA reverse transcription kit (Applied Biosystems, Waltham, MA) according to the manufacturer’s instructions. RT-qPCR was performed using an Applied Biosystems QuantStudio 6 Pro Real-Time PCR system and PowerUp SYBR green master mix (Thermo Fisher Scientific). Primers that spanned an intron-exon junction when possible were designed using the primer-BLAST program (https://www.ncbi.nlm.nih.gov/tools/primer-blast/). All primers were made by IDT (Coralville, IA). The sequences of the primers are shown in Table 2.

**TABLE 2:** Primers used for RT-qPCR. All sequences are given in the 5′ to 3′ direction.

Gene	Reverse sequence	Forward sequence
HPRT1	GGT​CCT​TTT​CAC​CAG​CAA​GCT	TGA​CAC​TGG​CAA​AAC​AAT​GCA
ITGB3 (β3 integrin)	TTC​TTC​GAA​TCA​TCT​GGC​C	GTG​ACC​TGA​AGG​AGA​ATC​TGC
ITGA5 (α5 integrin)	GTGGCCACCTGACGCTCT	TGC​AGT​GTG​AGG​CTG​TGT​ACA
VIM	TAC​AGG​AAG​CTG​CTG​GAA​GG	ACC​AGA​GGG​AGT​GAA​TCC​AG
SNAI1	TGCGTCTGCGGAACCTG	GGA​CTC​TTG​GTG​CTT​GTG​GA
SNAI2	ACC​CCA​CAT​CCT​TCT​CAC​TG	CCGACAAGTGACAGCCAT
TWIST1	AAG​GCA​TCA​CTA​TGG​ACT​TTC	GCC​AGT​TTG​ATC​CCA​GTA​TTT​T
ACTA2 (αSMA)	GTG​TTG​CCC​CTG​AAG​AGC​AT	GCT​GGG​ACA​TTG​AAA​GTC​TCA

### Immunofluorescence microscopy

TM cells were plated onto coverslips pre-coated with 5 µg/mL plasma fibronectin or 40 µg/mL human type I collagen (Millipore Sigma) at a density of 3 × 10^4^ cells/well as previously described (Johns et al., 2025). After 3 h, cells were fixed with 2% paraformaldehyde in phosphate buffered saline (PBS) for 20 min and then permeabilized with 0.1% Triton X-100 in PBS for 10 min. The cells were blocked with 1% BSA in PBS for 30 min, labeled with mouse primary monoclonal antibodies [BV3] (Abcam, #ab7166; RRID: AB_305742) at 1 µg/mL or LIBS2 (Millipore-Sigma, #MABT27; RRID: AB_10806476) at 10 µg/mL for 1 h at room temperature. The primary antibodies were detected using a 1:500 dilution of Alexa 546-conguated goat anti-mouse IgG (ThermoFisher Scientific). A 1:300 dilution of Alexa 488-conjugated phalloidin was used during this step to detect F-actin (ThermoFisher Scientific). Hoechst 33342 at 1 µg/mL was used to label nuclei. Coverslips were mounted onto slides using Shandon™ Immu-mount (ThermoFisher Scientific) and cells were imaged using a Zeiss Imager M2 fluorescence microscope together with the Zen image acquisition software version 3.079.

To detect αSMA, TM cells were fixed and permeabilized with ice-cold methanol for 15 min at −20 °C and then blocked in 1% BSA in PBS (1% BSA/PBS) for 30 min at room temperature. Fixed cells were labeled with rabbit anti-α-SMA antibody (Abcam, #ab5694; RRID: AB_2223021) at 2 µg/mL for 1 h at room temperature followed by a 1:500 dilution of Alexa 546-conguated goat anti-rabbit IgG for 30 min at room temperature. Cells were labeled with Hoechst 33342 to detect nuclei. Coverslips were mounted and imaged as described above. Relative fluorescence intensity was measured using Zeiss software (Zen version 3.079).

### Flow cytometry

Levels of α5, αvβ3 and active αvβ3 integrin on the TM cell surface were measured as previously described (Johns et al., 2025). Briefly, TM cells were lifted with Cell Dissociation Solution Non-enzymatic (Sigma-Aldrich Corp.), blocked for 30 min on ice with 1% BSA in PBS and labeled for 1 h on ice with 10 μg/mL α5 (P1D6, ThermoFisher Scientific, #12-4900-42, RRID:AB_10717080), total αvβ3 (LM609, Sigma-Millipore, mAb 1976, RRID:AB_2296419), and active αvβ3 (LIBS2, Millipore Sigma, MABT27, RRID:AB_10806476) integrin antibodies in 1% BSA/PBS. This was followed by a secondary Alexa 647-conjugated rabbit anti-mouse IgG (Thermo Fisher Scientific) diluted 1:400 in 1% BSA/PBS for 45 min on ice. Labeled cells were washed, resuspended in 1%BSA/PBS, strained with a 40–70 μm strainer to remove clumps and analyzed with the Cytek NL-3000 flow cytometer (Cytec Biosciences, Fremont, CA, USA) and the SpectroFlo program. Flow-Jo version 10.1 was used to analyze and graph the results. Unlabeled cells in 1% BSA/PBS were used as a control for autofluorescence.

### Immunolabeling of anterior segments for αSMA and α5 integrin

Wedges of anterior segments were cut, fixed with 4% paraformaldehyde in PBS, and embedded in paraffin as previously described (Filla et al., 2023). None of the donor tissues had a history of glaucoma. Sagittal tissue sections 5 µm thick were cut and deparaffinized in xylenes and rehydrated through a series of 100%–50% ethanol solutions. For αSMA labeling, antigen retrieval was performed on the sections using 0.05% trypsin in PBS at 37 °C for 20 min. For α5 integrin labeling, 95 °C antigen retrieval was performed using R-Universal epitope recovery buffer (Electron Microscopy Sciences, Hatfield, PA). Sections were blocked with 1% BSA in PBS and labeled overnight at 4 °C with 1 µg/mL mouse anti-αSMA monoclonal antibody ASM-1 (Sigma-Millipore, #CBL 171; RRID: AB_2223166) or 4 µg/mL mouse anti-α5 integrin monoclonal antibody 10F6 (ThermoFisher Scientific, #MA5-15568; RRID:AB_10979290). Sections labeled with either 1 or 4 µg/mL mouse anti-β-galactosidase monoclonal antibody GAL-13 (Sigma-Millipore, #G8021; RRID:AB_259970) were used as negative controls to confirm specificity of the labeling for αSMA and α5 integrin, respectively. The primary antibodies were detected using a 1:500 dilution of Alexa 546-conjugated goat anti-mouse IgG (ThermoFisher, Scientific, #A-11030). Nuclei were labeled with Hoechst 33342 at 1 µg/mL. Sections were mounted onto glass coverslips using Shandon™ Immu-mount. Labeled sections were imaged as described above.

### Lentiviral shRNA knockdown of α5 or β3 integrin

TM cells were plated at a density of 5 × 10^4^ cells/2.8 cm^2^ and grown to 70%–80% confluency. Cells were then transduced using a MOI of 50, 100 or 150 with either a α5 integrin shRNA lentiviral vector (MISSION® lentiviral particles clone ID TRCN0000029653, Sigma-Aldrich), or β3 integrin shRNA lentiviral vector (Mission® lentiviral particles clone ID TRCN0000003235, Sigma-Aldrich). Non-targeting lentiviral particles (MISSION®lentiviral particles #SHC016VN, Sigma-Aldrich) as well as untransduced cells were used as controls. These controls are referred to as Con-NT and Con-UT, respectively. Twenty-four hours later, transduced cells were selected using 1.5 mg/mL G418 sulfate (Corning, 61-234-RG). Transduced cells were maintained under selection for the duration of the experiments. Seventy-two hours after transduction, some transduced cells were harvested for RNA and used for RT-qPCR analysis as described above. RT-qPCR was performed using primers against α5 integrin, β3 integrin, αSMA, and HPRT1 for the housekeeping gene (Table 2). Cells that were not transduced were also used as a control.

### Western blot analysis

Six days after transduction, TM cells were lysed with 25 mM HEPES, pH 7.4 buffer containing 150 mM NaCl, 1 mM EDTA, 1 mM NaF, 1% NP-40, 0.25% deoxycholate, HALT phosphatase inhibitor cocktail, and HALT protease inhibitor cocktail (Thermo Fisher Scientific, Inc.) as previously described (Faralli et al., 2013; Johns et al., 2025). Cell lysates were clarified by centrifugation at 10,000 × g for 10 min at 4 °C and run on 10%-SDS-PAGE gels. Proteins were then transferred to a nitrocellulose membrane (Bio-Rad Laboratories). The membranes were blocked overnight at 4 °C with 3% BSA in 20 mM Tris pH 7.4, 150 mM NaCl (TBS) buffer and then incubated with a primary antibody in 1% BSA/TBS/0.1% Tween-20 for 1 h at room temperature. Primary antibodies used were rabbit anti-α5 integrin (Cell Signaling Technology, Cat # 4705S, RRID:AB_2233962, 1:1000), rabbit anti-αSMA (Abcam, #ab5694; RRID: AB_2223021), 1:250, rabbit anti-β3 integrin (Cell Signaling Technology clone D7X3P, cat #13166S; RRID:AB_2798136, 1:1000) and rabbit anti-GAPDH (Abcam cat # 9485; RRID: AB_307275, 1:2500). Membranes were washed with TBS/0.1% Tween-20 and incubated for 1 h with a secondary antibody (LI-COR Biosciences, Lincoln, NE, USA, IRDye 800CW goat α-rabbit or mouse, 1:15000). Labeled bands were visualized using a LI-COR Odyssey scanner and quantified using LI-COR Image Studio v. 5.0.21 software (LI-COR Biosciences). GAPDH or a revert 700 Total Protein Stain (Li-Cor) were used as loading controls.

### Data analysis

Data were presented as the mean ± SEM. Statistical comparisons used either a t-test (Graphpad https://www.graphpad.com/quickcalcs/ttest1/) or a one-way ANOVA plus the *post hoc* Tukey HSD test (https://astatsa.com/OneWay_Anova_with_TukeyHSD/). An on-line statistical calculator was used to determine the significance of the slopes in the Pearson coefficient plots (www.socscistatistics.com/pvalues/pearsondistribution.aspx). A *p*-value < 0.05 was considered statistically significant. The specific tests used are described within each figure legend. Fold changes in gene expression were performed according to the ΔΔCt method. The RT-qPCR data was normalized using either gene succinate dehydrogenase complex subunit A (SDHA), or hypoxanthine phosphoribosyl transferase 1 (HPRT1).

## Results

**FIGURE 1:**
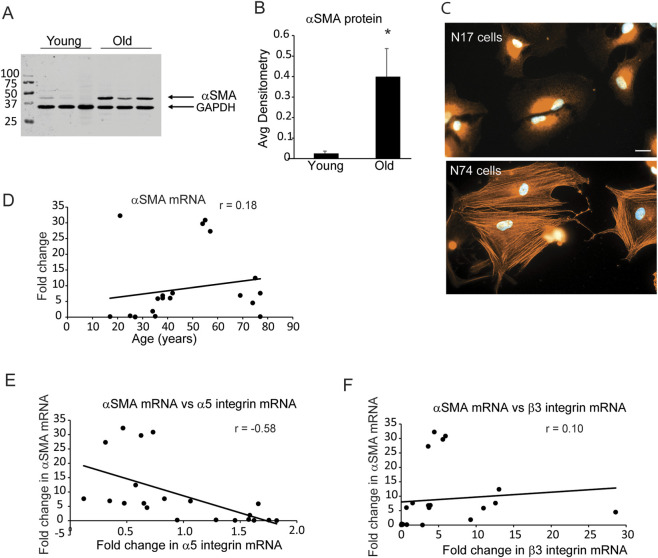
Young TM cells express lower αSMA levels than old TM cells. **(A)** Lysates were harvested from three TM cell strains (N25, N27, N27-2) derived from young donor eyes (ages 25 and 27) and three cell strains (N74, N75, N77) derived from old donor eyes (ages 74, 75, 77) 1 week after reaching confluency. Protein lysates from each culture (20 µg) were run on a 10% SDS-PAGE gel for Western blot analysis and probed with a rabbit anti-αSMA antibody. An anti-GAPDH antibody was used as a loading control. The blot is representative of experiments done in triplicate using biological replicates. **(B)** Densitometry was performed and αSMA levels were normalized to GAPDH levels. αSMA levels in young cells were significantly different from old cells, *p < 0.001. **(C)** Immunofluorescence micrographs of young (N17) and old TM (N74) cells obtained from a 17 and 74- year-old donor eyes plated onto 5 µg/mL fibronectin and labeled for αSMA. Only the N74 cells showed robust αSMA positive stress fibers. Scale bar = 20 µm. **(D)** Scatter plot comparing levels of αSMA mRNA relative to age. Although levels of αSMA mRNA appear to increase with age, this increase did not show a significant correlation with age. *N* = 21 cell strains, ages 17–77. **(E,F)** Scatter plots comparing αSMA mRNA levels relative to fold change in α5 and β3 integrin mRNA levels. Fold changes of αSMA mRNA levels showed an inverse correlation to fold changes in α5 integrin mRNA and decreased as α5 integrin mRNA levels increased. The correlation was statistically significant (*p* < 0.05) using a t-test. αSMA and β3 integrin mRNA did not show a correlation as β3 integrin mRNA increased. *N* = 21 cell strains, ages 17–77 (Johns et al., 2025). *r* = Pearson’s coefficient.

Previous studies have shown that TM cells isolated from older donor eyes (>70 years of age) that express lower levels of the α5β1 integrin (Johns et al., 2025) exhibited higher levels of αSMA and formed stress fibers containing αSMA. Western blot analysis and immunofluorescence microscopy confirmed these earlier studies. Figures 1A,B show that three TM cell strains from older donor eyes (74, 75 and 77 years old) expressed significantly higher protein levels of αSMA compared to three TM cell strains isolated from young donor eyes (25, 27 and 27 years old). Older TM cells also assembled more robust αSMA positive stress fibers. Figure 1C shows that TM cells isolated from a 17-year old normal (N17) donor eye did not show any αSMA positive stress fibers. In contrast, TM cells from a 74-year old normal (N74) donor eye contained αSMA positive stress fibers supporting the idea that αSMA is upregulated in older individuals. Interestingly, correlation coefficient analysis of the mRNA levels for αSMA did not show an age-related increase, since the Pearson’s coefficient was 0.18 (Figure 1D). This suggests that age was not the predominant driving factor in the upregulation of αSMA mRNA levels. Since the increase was most noticeable in cells that expressed lower levels of α5β1 integrin (Johns et al., 2025), we then compared fold changes in mRNA levels for αSMA with fold changes in the mRNA for the α5 integrin subunit (Figure 1E). The correlation coefficient plot had a Pearson’s coefficient of *r* = −0.58 suggesting that there was an inverse relationship between mRNA levels for αSMA and α5 integrin. Thus, the levels of mRNA for αSMA increased as the levels of mRNA for the α5 integrin subunit decreased. This correlation was specific for the α5 integrin subunit since the mRNA for the β3 integrin did not show a significant correlation with mRNA levels for αSMA (compare Figure 1E and Figure 1F).

**FIGURE 2:**
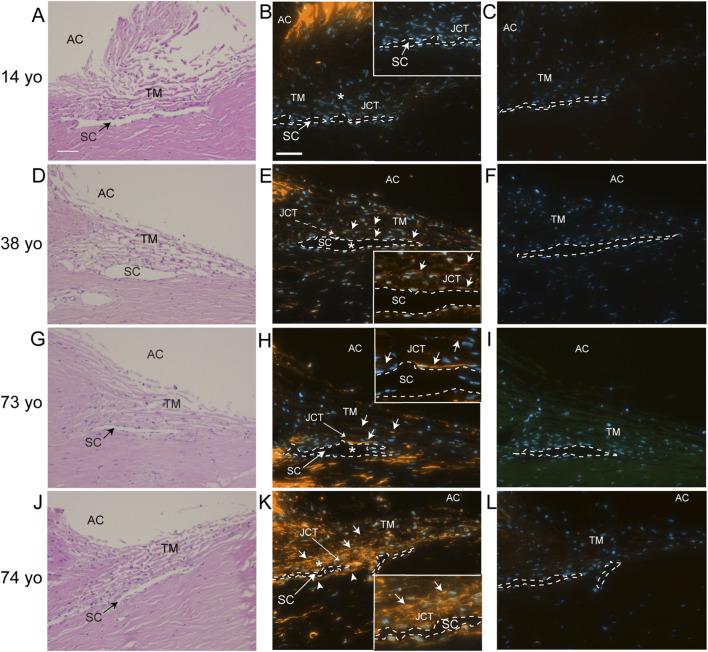
Immunolabeling for αSMA in human anterior segments from young and old eyes. **(A,D,G,J)** H&E staining of the TM/SC pathways of donors of 14, 38, 73, and 74 years of age, respectively. **(B)** αSMA labeling was essentially absent from the 14-year-old donor tissue. **(E,H)** Weak αSMA labeling (solid arrows) was observed in the trabecular beams, JCT and SC of the 38 and 73- year-old donor tissue, although strong labeling was observed in portions of the SC inner wall in the 73-year-old donor tissue. **(K)** Very strong αSMA labeling (solid arrows) was observed throughout much of the TM/SC pathway of the 74-year-old donor tissue including the JCT and SC. This includes the SC outer wall (arrowheads). **(C,F,I,L)** No labeling was observed in sections incubated with a control antibody against β-galactosidase. Nuclei are identified in sections labeled for αSMA or β-galactosidase using Hoechst 33342. AC, anterior chamber; TM, trabecular meshwork; SC, Schlemm Canal which is outlined with a dashed line and JCT, juxtacanalicular tissue. Asterisks in **(B,E,H,K)** indicate enlarged areas shown in the insets. Scale bar = 50 µm in **(A,B)**.

**FIGURE 3:**
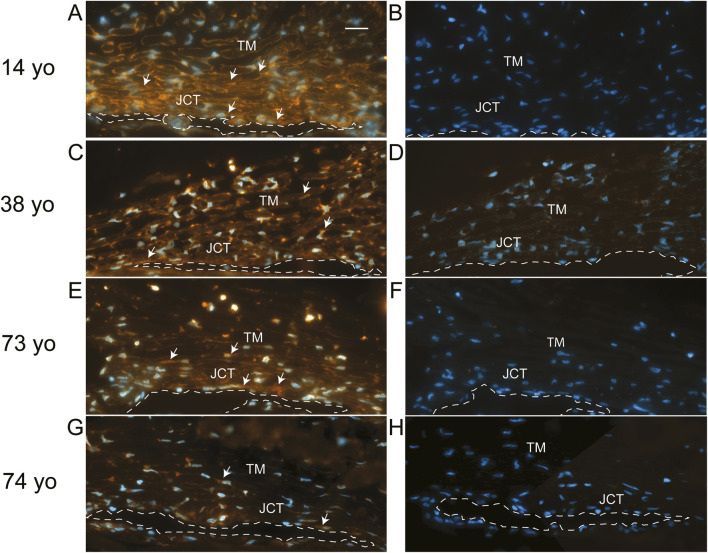
Immunolabeling for α5 integrin in human anterior segments from young and old eyes. Localization of α5 integrin in the trabecular meshworks of 14, 38, 73. and 74-year-old donor tissue used in Figure 2. **(A,C)** Integrin labeling intensity was relatively strong in the 14-year-old donor tissue and moderate in the 38-year-old donor tissue, respectively. α5 integrin was localized in the cells found on the trabecular beams and in the JCT of the TM and in the SC endothelial cells of both tissue samples. **(E)** Weak to moderate α5 integrin labeling intensity was also observed in the TM/SC in the 73- year-old tissue. **(G)** Weak α5 integrin labeling was observed in the TM/SC from the 74-year-old donor. **(B,D,F,H)** No labeling was observed in sections from the same donor tissues incubated with a control antibody against β-galactosidase. Arrows = α5 integrin-positive cells. TM, trabecular meshwork; SC, Schlemm Canal which is outlined with a dashed line; JCT, juxtacanalicular tissue. Scale bar in panel A = 20 µm.

To see if elevated levels of αSMA correlated with TM cells lacking or expressing low levels of α5 integrin expression *in vivo*, human anterior segments from young and old donor eyes were labeled for αSMA (Figure 2) and the α5 integrin subunit (Figure 3). As shown in Figures 2B,E,H, TM cells in tissues from 14, 38, and 73-year-old individuals showed weak αSMA labeling in the JCT region of the TM adjacent to SC in the anterior chamber (AC) compared to TM cells in the JCT of the anterior segment from a 74-year-old individual (Figure 2K). Analysis of the α5 integrin levels in those same tissues showed that, unlike the tissues from the 14, 38- and 73-year-old individuals, the TM and SC from the 74-year-old which contained high levels of αSMA (Figure 2K) had very low levels of α5β1 integrin (Figure 3G). This suggests that there may be an inverse correlation between αSMA and α5 integrin expression *in vivo*.

**FIGURE 4:**
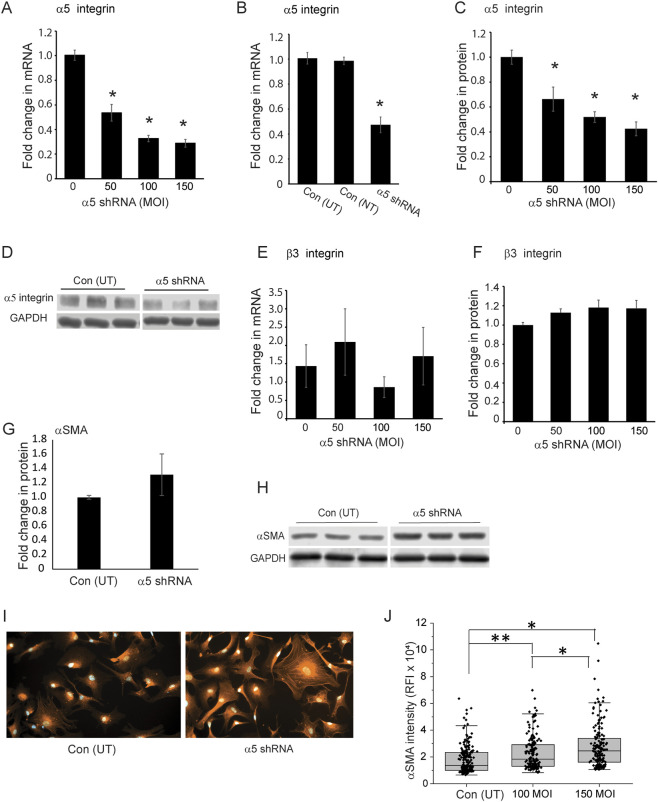
Knockdown of α5 integrin subunit in young TM cells triggers an increase in αSMA expression. **(A)** Cells isolated from 25 and 35‐year‐old donor eyes were transduced with increasing MOIs of α5 integrin shRNA lentiviral particles (MOI 50, 100, 150). By RT-qPCR, there was a significant 60% reduction in α5 integrin mRNA at the 100 and 150 MOIs compared to cells not transduced (0). **(B)** TM cells transduced with non-targeting control lentiviral particles (Con-NT) showed no statistical difference in the mRNA levels for the α5 integrin compared to untransduced control cells (Con-UT). In contrast, cells transduced with α5 integrin shRNA lentiviral particles (MOI 100) showed a statistically significant 50% decrease in the mRNA levels for the α5 integrin. **(C)** A significant reduction in protein levels was also observed at all MOIs used. The α5 integrin protein levels were normalized to GAPDH which was used as a loading control. **(D)** Representative SDS-PAGE of α5 integrin levels obtained from N35 cells transduced at a MOI of 100 and untransduced control cells. Experiments were done in triplicates using two biological replicates (N25 and N35 cells). **(E,F)** Levels of mRNA and protein for the β3 integrin were unaffected by α5 integrin knockdown at any MOI used. **(G)** Densitometry of western blots of cell lysates from N25 and N35 cells showed αSMA protein levels were elevated in cells transduced at a MOI of 100. αSMA levels were normalized to GAPDH levels. **(H)** Representative Western blot of αSMA levels in transduced and non-transduced N35 cells. Blots were down in triplicate using biological replicates of each cell strain and repeated twice. **(I)** Representative images of immunofluorescence labeling of untransduced and transduced N35 TM cells for αSMA showed that the level of αSMA intensity varied between the cells but was increased in α5 integrin shRNA transduced cells. The variation in cell spreading could be due to the fact that cells were not synchronized prior to the start of the spreading assay. **(J)** Relative mean fluorescence intensity (RFI) of total αSMA labeling in non-transduced and transduced N25 and N35 cells plated on collagen. Transduced cells show a statistically significant increase in αSMA labeling compared to non-transduced cells. *N* = 40 cells per treatment group. *p <0.001, **p < 0.003 Experiments were done in triplicates. Scale bar = 20 µm. Data for RT-qPCR, densitometry and RFI studies were pooled data from N25 and N35 cells.

Since integrins have been shown to control actomyosin stress fiber formation (Burridge and Chrzanowska-Wodnicka, 1996; Schoenwaelder and Burridge, 1999), we wanted to determine whether αSMA expression was dependent on α5β1 integrin expression. To test this, we used α5 shRNA lentiviral particles to knockdown expression of α5β1 integrin in cells from two young normal donor eyes ages 25 (N25) and 35 (N35) to see if this would lead to an increase in αSMA levels. Figure 4A shows that transducing cells with 100 or 150 MOI of α5 integrin shRNA lentiviral particles resulted in a statistically significant (*p* < 0.001) 60% knockdown in α5 integrin mRNA compared to control cells not transduced (0; Con UT) with the α5 shRNA lentiviral particles. To further demonstrate specificity of the knockdown, we transduced cells with a non-targeting shRNA lentiviral vector (Con NT). As shown in Figure 4B, non-targeting shRNA lentiviral particles had no effect on the levels of α5 integrin mRNA compared to untransduced control cells (Con UT). Western blot analysis of the cells transduced with either 100 or 150 MOI of the α5 integrin shRNA lentiviral particles verified the specificity of the knockdown and showed that the knockdown resulted in a statistically significant (*p* < 0.001) 40% decrease in the protein levels of α5 integrin (Figures 4C,D). To further demonstrate specificity of the knockdown, we looked to see if the knockdown of α5 integrin affected the level of the β3 integrin. As shown in Figures 4E,F, knockdown of α5 integrin mRNA and protein levels did not result in a significant change in either β3 integrin mRNA or its protein levels, even when higher MOIs of the α5 integrin shRNA lentiviral particles were used suggesting that the α5 shRNA was specific for α5 integrin subunit.

Knocking down expression of the α5 integrin subunit, however, did result in an increase in the expression of αSMA protein levels in cells transduced with the α5 integrin shRNA lentivirus (Figures 4G–J). Western blot analysis (Figures 4G,H) showed that there was a modest increase in αSMA in the α5 integrin shRNA transduced cells compared to control untransduced cells when the levels were normalized to the GAPDH control, although this increase was not statistically significant. This increase, however, was supported by immunofluorescence microscopy studies that showed a statistically significant increase in the intensity of αSMA expression in transduced cells (Figures 4I,J) at both a MOI of 100 (*p* < 0.003) and 150 (*p* < 0.001) compared to the non-transduced cells. Figure 4I shows representative images of αSMA labeling in non-transduced and transduced N35 cells.

**FIGURE 5:**
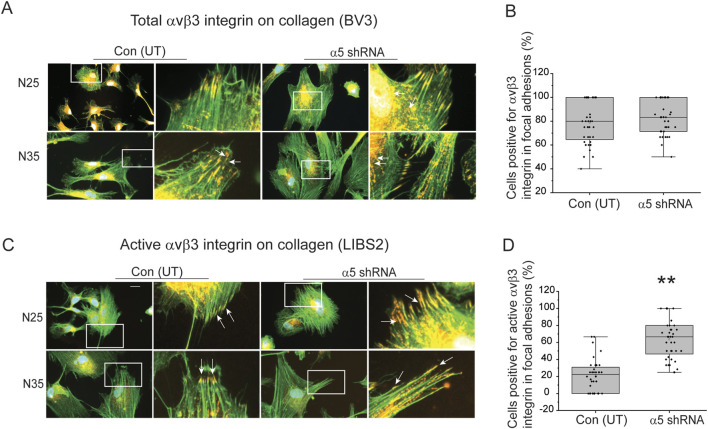
Knockdown of a5 integrin subunit triggers an increase in αvβ3 integrin activity in focal adhesions in young TM cells on collagen. **(A)** Cells labeled with Alexa 488 conjugated phalloidin (green) to localize actin filaments and with mAb [BV3] (red) show both transduced and untransduced TM cells plated on collagen contained numerous focal adhesions (white arrows) containing αvβ3 integrin. **(B)** Quantitation of percentage of untransduced and transduced cells containing three or more focal adhesions with αvβ3 integrin. No statistical difference in the percentage of cells that contained total αvβ3 integrin in focal adhesions was detected. **(C)** Cells were labeled for active αvβ3 integrin (mAb LIBS2, red) in focal adhesions and actin stress filaments (Alexa 488-phalloidin, green) in transduced and untransduced N25 and N35 cells plated on collagen. More transduced TM cells contained focal adhesions (white arrows) with active αvβ3 integrin than untransduced cells. **(D)** Quantitation of the percentage of untransduced and transduced N25 and N35 cells containing three or more focal adhesions with active αvβ3 integrin. Transduced TM cells showed a statistically significant (*p* < 0.001) difference in the percentage of cells that contained active αvβ3 integrin in focal adhesions. Scale bar = 20 µm. Experiments were done in triplicates using two biological replicates. *N* = 40 cells.

As expected from our previous studies (Johns et al., 2025), the increase in the intensity of αSMA expression correlated with increased levels of activated αvβ3 integrin in focal adhesions. Using the same cells shown in Figure 4, Figures 5A,B show that knockdown of the α5 integrin subunit did not affect the percentage of N25 and N35 cells expressing total αvβ3 integrin in focal adhesions plated on collagen. However, the knockdown of α5 integrin subunit did cause a statistically significant (*p* < 0.001) increase in the percentage of N25 and N35 cells containing active αvβ3 integrins in focal adhesions (Figures 5C,D). This increase in activated levels of αvβ3 integrin corresponded to an increase in αSMA intensity in the N25 and N35 cells by immunofluorescence microscopy (Figure 4J).

**FIGURE 6:**
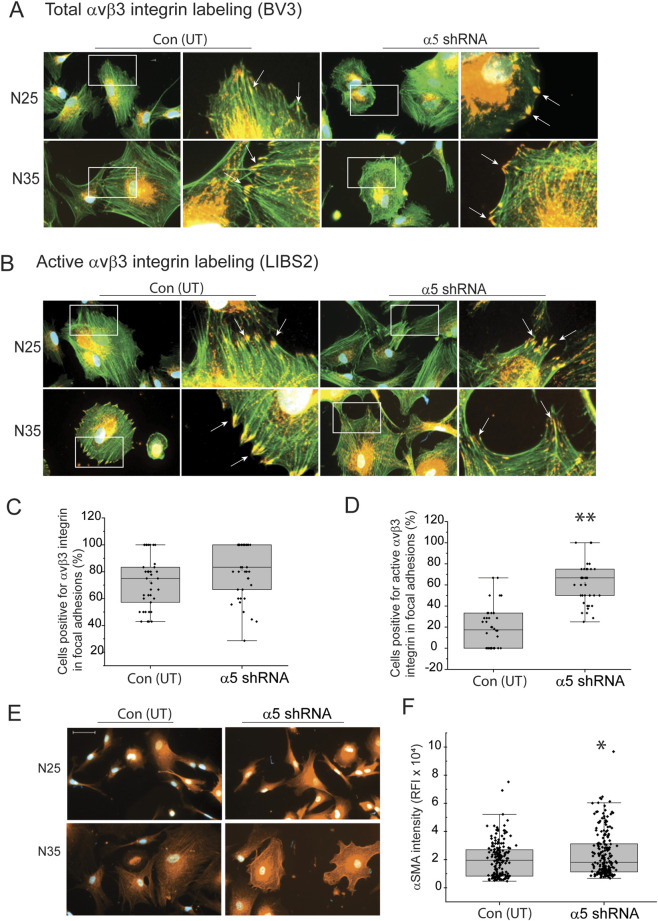
Knockdown of a5 integrin subunit triggers an increase in αvβ3 integrin activity in focal adhesions in young TM cells on fibronectin. **(A)** Immunolabeling for total αvβ3 integrin (mAb [BV3], red) in focal adhesions and actin filaments with Alexa 488-phalloidin (green) in transduced and untransduced N25 and N35 cells plated on fibronectin. Both transduced and untransduced TM cells contained numerous focal adhesions (white arrows) containing αvβ3 integrin. **(B)** Immunolabeling for active αvβ3 integrin (mAb LIBS2, red) in focal adhesions and actin filaments (Alexa 488-phalloidin, green) in transduced and untransduced N25 and N35 cells plated on fibronectin. More untransduced TM cells contained focal adhesions (white arrows) containing active αvβ3 integrin. **(C)** Quantitation of percentage of untransduced and transduced N25 and N35 cells containing three or more focal adhesions with αvβ3 integrin. No statistical difference in the percentage of cells that contained total αvβ3 integrin in focal adhesions was detected. Experiments were done in duplicates using two biological replicates. *N* = 40 cells. **(D)** Quantitation of the percentage of untransduced and transduced cells containing three or more focal adhesions with active αvβ3 integrin. Transduced TM cells showed a statistically significant increase in the percentage of cells that contained active αvβ3 integrin in focal adhesions. *N* = 40 cells. **(E)** Representative immunofluorescence images of untransduced and transduced N25 and N35 TM cells plated on fibronectin and labeled for αSMA. Images show that the αSMA intensity appears to be greater in transduced cells. Scale bar = 20 µm. **(F)** Relative mean fluorescent intensity (RFI) of total αSMA labeling in untransduced and transduced N25 and N35 cells. Transduced cells show a statistically significant increase in αSMA labeling intensity compared to non-transduced cells. *N* = 40 cells.

We then repeated the study to see if we saw similar results when we plated the α5 integrin knockout TM cells on fibronectin to determine if the substrate cells were plated on could affect the expression of αSMA. As shown in Figures 6A,C, there was no significant difference in total αvβ3 integrin-positive focal adhesions between transduced and non-transduced N25 and N35 cells plated on fibronectin. In contrast, the knockdown of α5 integrin subunit in both N25 and N35 cells resulted in a statistically significant increase (*p* < 0.001) in the percentage of cells containing activated αvβ3 integrin in focal adhesions (Figures 6B,D). The increase in activated αvβ3 integrin correlated with an increase in the intensity of αSMA labeling in transduced cells compared to non-transduced cells (Figures 6E,F). This suggests that the increase in activated αvβ3 integrin and αSMA levels were not dependent on the substrate. Interestingly, whether the cells were plated on collagen or fibronectin, we rarely saw well-formed robust αSMA containing stress fibers in these transduced young cells like the ones we saw in older TM cells (compare Figure 1C to Figure 4I). This suggests that although activated levels of β3 integrin are associated with the expression of αSMA, some other factor(s) such as the expression of a specific tropomyosin isoform may be needed for the robust assembly of αSMA into the stress fibers seen in older cells (Prunotto et al., 2015).

**FIGURE 7:**
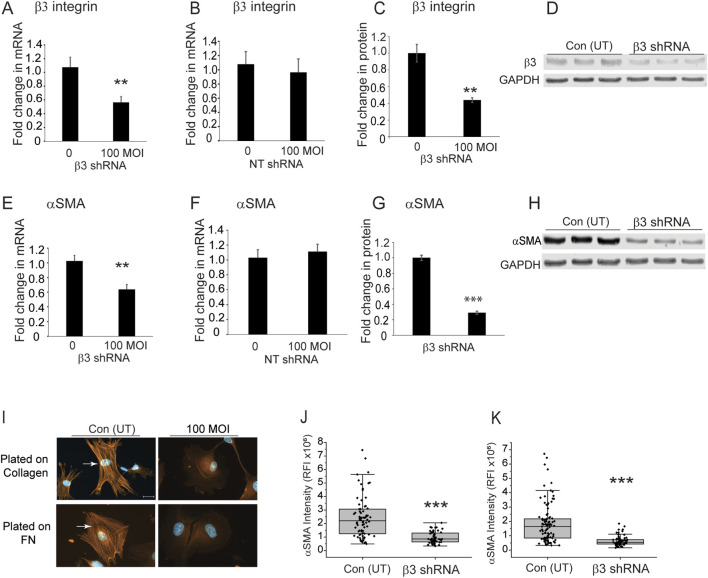
Knockdown of β3 integrin subunit in TM cells from a 77 year-old triggers a decrease in αSMA levels. **(A)** N**77** TM cells were transduced with β3 shRNA lentiviral particles (MOI 100). By RT-qPCR, there was a significant 40% reduction in β3 integrin mRNA compared to untransduced cells (**p < 0.008). **(B)** N77 TM cells transduced with a non-targeting lentiviral vector showed no statistically significant difference in β3 integrin mRNA levels compared to untransduced cells. **(C)** Densitometry of Western blot analyses of β3 integrin protein levels also showed a statistically significant 60% decrease (**p < 0.006) in β3 integrin protein levels compared to untransduced cells. **(D)** Representative western blots of cell lysates from transduced and untransduced cells. GAPDH was used as a loading control. **(E)** RT-qPCR showed αSMA mRNA levels were significantly reduced (**p < 0.002) by 40% in transduced cells. **(F)** TM cells transduced with non-targeting lentivirus particles showed no statistically significant difference in αSMA mRNA levels compared to untransduced cells. **(G)** Densitometry of western blots showed that protein levels were also significantly reduced (***p < 0.0006) in cells transduced at a MOI of 100 compared to untransduced cells. αSMA levels were normalized to GAPDH levels. **(H)** Representative Western blot of αSMA levels in transduced and untransduced cells. **(I)** Immunolabeling studies showed that transduced cells plated on collagen or fibronectin had no αSMA-positive stress fibers. In contrast, untransduced cells contained αSMA-positive stress fibers. **(J,K)** Relative fluorescence intensity of αSMA labeling in untransduced and transduced cells plated on either collagen **(J)** or fibronectin **(K)**. Transduced cells show a statistically significant decrease (***p < 0.0006) in αSMA labeling intensity. Scale bar = 20 µm. All experiments were done in triplicates using technical replicates from independent experiments.

To see if β3 integrin was involved in the expression of αSMA, we then used shRNA lentiviral particles to knockdown expression of the β3 integrin subunit in TM cells isolated from a normal donor eye age 77 (N77). These older TM cells, which form αSMA positive stress fibers, had previously been shown to contain high levels of activated αvβ3 integrin and expressed very low levels of α5β1 integrin (Johns et al., 2025). As shown in Figure 7A, a MOI of 100 resulted in a knockdown of the β3 integrin mRNA by 40% (*p* < 0.008) compared to untransduced control cells. In contrast, transduction with non-targeting lentiviral particles had no effect on β3 integrin mRNA levels (Figure 7B). As shown in Figures 7C,D, knockdown of the β3 integrin mRNA resulted in a 60% decrease (*p* < 0.006) in protein levels. When we looked at the levels of αSMA in the β3 integrin knockdown cells compared to untransduced cells, we saw that the levels of *αSMA* mRNA and protein levels were significantly reduced by 40% (*p* < 0.002) and 60% (*p* < 0.00006), respectively (Figures 7E,G,H). As expected, transduction with the non-targeting lentiviral vector had no statistical effect on *αSMA* mRNA levels (Figure 7F). By immunofluorescence microscopy, we saw a statistically significant (*p* < 0.00006) decrease in αSMA in the cytoplasm of cells regardless of whether they were plated on collagen or fibronectin coated coverslips (Figures 7I–K). This suggests that expression of αvβ3 integrin affects the mRNA and protein levels for αSMA in TM cells and hence, it may be involved in the transition of TM cells into a mesenchymal phenotype since αSMA is considered an early biomarker for this transition.

**FIGURE 8:**
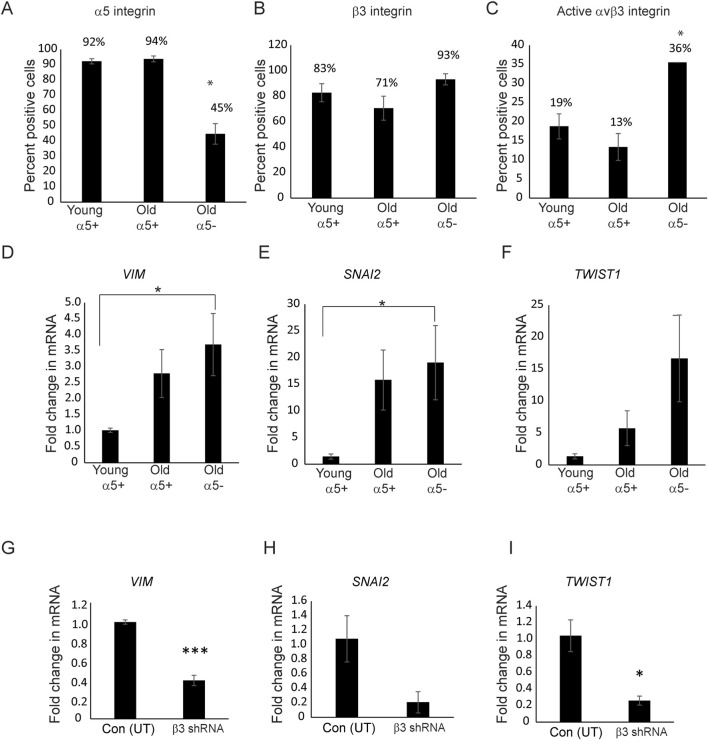
Activity of *αvβ3* integrin affects the expression of *VIM*, *SNAI2*, and *TWIST1* mRNA levels. **(A)** Flow cytometry showed that TM cells (Old α5-) derived from 74 to 77-year-old donor eyes expressed lower levels of α5 integrin compared to TM cells derived from young (young α5+) and other old donor eyes (old α5+). The designation α5+ refers to the fact that a large percentage of cells express the α5 integrin subunit while α5- refers to the fact that most of these cells do not express the α5 integrin subunit. **(B)** Despite differences in the levels of the α5 integrin subunit, all three populations of cells expressed similar levels of β3 integrin. **(C)** More old a5- TM cells (N74 and N77) expressed active αvβ3 integrin on the cell surface compared to young and old TM cells expressing α5β1 integrins; *p < 0.02. *N* = 10,000 cells per condition. *N* = 7 young α5+ biological replicates (ages 17–36), *N* = 5 old α5+ biological replicates expressing α5β1 integrin (ages 55–75). *N* = 2 old α5- biological replicates (ages 74–77). Cells were labeled with P1D6 (α5β1 integrin), LM609 (total αvβ3 integrin), or LIBS2 (active αvβ3 integrin) mAbs. **(D,E)** RT-qPCR showed that Old α5- TM cells expressed significantly higher levels (*p* < 0.04) of mRNA for the EndMT markers *VIM* and *SNAI2* compared to cells isolated from young α5+ donor eyes (N25 and N35).**(F)** *TWIST1* mRNA levels were also higher in the old α5 integrin negative cells but the levels were not statistically significant (*p* < 0.08). **(G–I)** Knockdown of β3 integrin using shRNA in the old N77 cells (Old α5-) that contained elevated levels of active αvβ3 integrin and low levels of α5β1 integrin mRNA had statistically reduced levels of *VIM* (*p* < 0.0004) and *TWIST1* (*p* < 0.01) mRNA. Levels of *SNAI2* mRNA levels were also reduced, but not statistically (*p* < 0.07). All experiments were done in triplicates and repeated twice.

We then examined whether other biomarkers involved in EndMT might be affected by changes in the expression and/or activity of α5β1 and αvβ3 integrins in TM cells. Other biomarkers for a mesenchymal phenotype in EndMT that we investigated were SNAI1, SNAI2, Vimentin, TWIST1, and TWIST2 (Lamouille et al., 2014; Lovisa et al., 2020). SNAIs and TWISTs are transcription factors whose expression occurs early at the onset of EndMT and play a central role in driving EndMT, whereas vimentin is an intermediate filament that is upregulated in mesenchymal cells (Islam et al., 2021). As shown in Figures 8A–C, we used three populations of TM cells for the study. The first population of TM cells were obtained from donor eyes (ages 17–36 years old). Flow cytometry studies showed that the majority of the young cells expressed α5β1 (92%) and αvβ3 (94%) integrins on their cell surface (Figures 8A,B). However, as shown in Figure 8C, very few of these cells (only 19%) expressed the activated form of αvβ3 integrin on their cell surface. We called these cells young α5+ cells. The second group of TM cells which we called old α5 integrin+ were from older individuals (ages 55–75). Like the young α5+ cells, a large percentage of these cells expressed α5β1 (94%) and αvβ3 (71%) integrins on their cell surface and only a few expressed the active form of αvβ3 integrin (13%). The third population of the TM cells that we called old α5- were also from older normal donor eyes (ages 74 and 77). However, their integrin profile differed significantly (*p* < 0.02) from the other cell strains. Less than half the population expressed the α5β1 integrin (45%) on their cell surface and nearly all of the cells (93%) expressed αvβ3 integrin. In addition, a greater percentage of the old α5-cells (36% compared to 19% or 13%) expressed the activated form of αvβ3 integrin on their cell surface.

When we then compared the levels of EndMT biomarkers in the old α5-cells that expressed low levels of the α5 integrin subunit to the α5+ TM cells. As shown in Figures 8D–F, we found that the old α5- cells expressed statistically (*p* < 0.04) higher levels of both *VIM* and *SNAI2* mRNA compared to the young α5+TM cells. The *TWIST1* mRNA levels also appeared to be higher in these old α5-TM cells, but the increase was not statistically significant (*p* < 0.08). Interestingly, the levels of *VIM*, *SNAI2*, and *TWIST1* mRNA also appeared to be higher in the old α5+TM cells that express high levels of α5β1 integrin. However, this increase in *VIM*, *SNAI2*, and *TWIST1* mRNA in these old α5+ positive cells was not statistically significant. *SNAI1* and *TWIST2* mRNA were not detected in any of these cells. Hence, these studies suggest that as α5 integrin levels decrease and the level of activated αvβ3 integrin increases, the expression of these mesenchymal markers for EndMT are affected.

Since the knockdown of αvβ3 integrin appeared to reverse the effect on αSMA caused by the decrease in α5β1 integrin (Figure 7), we then looked at the mRNA levels for *VIM*, *SNAI2*, and *TWIST1* in the β3 shRNA lentiviral transduced old α5-TM cells as well (Figures 8G–I). As was seen for *αSMA* mRNA levels (Figure 7E), we saw a statistically significant decrease in *VIM* (*p* < 0.0004) and *TWIST1* (*p* < 0.02) mRNA levels in the αvβ3 integrin knockdown cells compared to untransduced cells. *SNAI2* mRNA levels were also lower in these knockdown cells, however, the reduction was not statistically significant (*p* < 0.08). Together these results suggest that expression of some EndMT biomarkers may be influenced by the expression levels of α5β1 integrins and the activity of αvβ3 integrins.

## Discussion

In this study, we show that changes in integrin expression contribute to the development of a mesenchymal phenotype in TM cells. This process which is commonly known as integrin switching (Madamanchi et al., 2014) resulted in a decrease in α5 integrin mRNA levels and the subsequent activation of αvβ3 integrin. Together these changes contribute to an increase in both αSMA mRNA and proteins levels and the assembly of αSMA into stress fibers, an early marker of EMT. We also saw that the expression of other mesenchymal biomarkers such as vimentin, SNAI2 and TWIST1 were also affected by this switch in integrin expression. Together these studies suggest that the increase in αvβ3 integrin activity caused by a decrease in α5β1 integrin expression may be an early step in the development of mesenchymal phenotype in TM cells.

Although changes in integrin expression have been known to play a role in the development of an EMT/EndMT phenotype in multiple tissues (Piera-Velazquez and Jimenez, 2019; Galliher and Schiemann, 2006; Parvani et al., 2015), this is the first study to suggest that the ratio between active αvβ3 integrin levels and α5β1 integrin expression may control the expression of early markers of EMT/EndMT. Specifically, we found that when the protein and mRNA levels of α5β1 integrin are low and the levels of active αvβ3 integrin are high, we saw an increase in the mRNA levels for EndMT markers *αSMA*, *VIM*, *SNAI2* and *TWIST1*. In contrast, when we lowered the levels of β3 integrin with shRNA in old TM cells, we triggered a decrease in *αSMA*, *VIM*, *SNAI2* and *TWIST1* mRNA expression. Since the expression of these markers defines the beginning of the phenotypic transition to myofibroblasts and the mesenchymal phenotype (Hinz et al., 2012; Jones and Ehrlich, 2011), this suggests that the activation levels of αvβ3 integrin relative to α5β1 integrin levels is an early event in the mesenchymal transition of a TM cell into a myofibroblast. In addition, since these biomarkers have previously been reported to be upregulated in glaucomatous cells, this suggests that integrin switching may be involved in the development of glaucoma (Yang et al., 2025).

**FIGURE 9:**
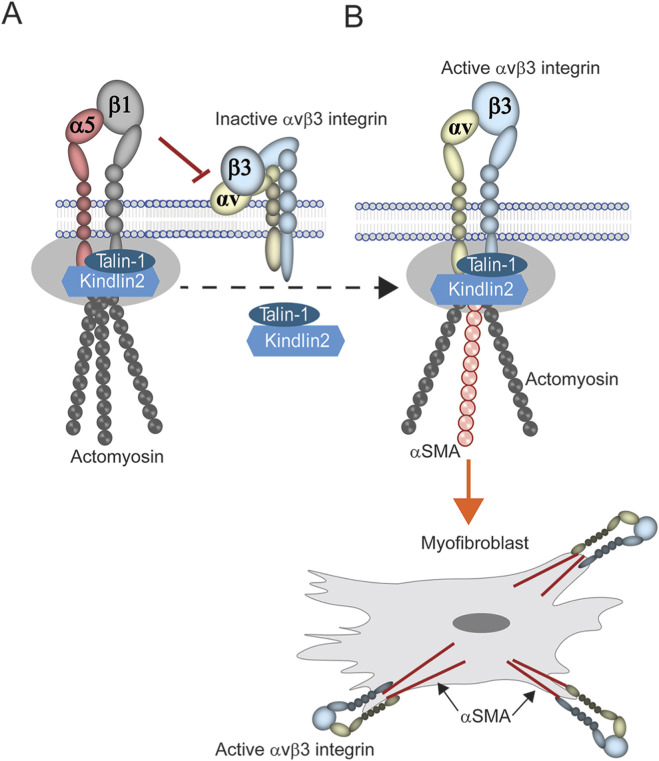
Model of transdominant inhibition of integrin signaling. **(A)** Expression of α5β1 integrin is activated when cytoplasmic proteins such as talin-1 and kindlin-2 bind to its cytoplasmic tails in focal adhesions, triggering the formation of the actomyosin network. Presumably, this leaves those proteins unavailable to bind to αvβ3 integrin thus leaving it inactive on the cell surface. **(B)** Loss of α5β1 integrin expression would free these proteins to bind and activate αvβ3 integrin. Since αvβ3 integrin appears to control αSMA expression, this in turn promotes the incorporation of αSMA into actomyosin networks and the development of the myofibroblast phenotype.

It is not surprising that the increase in αvβ3 integrin activity was triggered by a decrease in α5β1 integrin expression. One plausible explanation for this is a cross-talk process called transdominant inhibition which can occur when two integrins compete for the same cytoplasmic proteins, such as talin-1 or kindlin-2, that activate integrins (Calderwood et al., 2004). In this scenario, expression of α5β1 integrin inhibits αvβ3 integrin because it out competes αvβ3 integrin for binding to talin-1 and kindlin-2 (Figure 9A). However, when the levels of the α5β1 integrin decreases, αvβ3 integrin is now able to bind talin-1 and kindlin-2 and be activated (Figure 9B) (Lu et al., 2022; Lolo et al., 2022; Bouvard et al., 2013; Moser et al., 2009). What causes the loss of α5β1 integrin is still unknown. Early studies in fibroblasts have suggested that a change in the expression of transcription factors that control α5β1 integrin expression may be responsible (Gingras et al., 2003; Gingras et al., 2009). Alternatively, activation of αvβ3 integrin could also be enhanced if the recycling of α5β1 integrins was impaired, thus leading to the rapid and prolonged expression of αvβ3 integrin on the cell surface (Caswell et al., 2009; Arjonen et al., 2012). These two possibilities are not necessarily mutually exclusive and both processes could be occurring in older tissues.

How activation of αvβ3 integrin enhances αSMA expression is unclear. αSMA expression in EMT/EndMT is usually thought to occur via enhanced TGFβ signaling. Hence, it is not surprising that αvβ3 integrin was found to be involved in the development of a mesenchymal phenotype. In TM cells, activation of αvβ3 integrin induces the expression of TGFβ2 mRNA and protein which is a potent inducer of EMT and EndMT (Filla et al., 2021). Furthermore, multiple studies show that a synergistic interaction between αvβ3 integrin and TGFβ signaling triggers the development of a mesenchymal phenotype in a number of different cell types and diseases. For example, this interaction has been shown to be involved in TGF-β-induced EMT in mammary epithelial cells (Galliher and Schiemann, 2006; Parvani et al., 2015) possibly through ECM driven αvβ3 integrin crosstalk with the TGFβ pathway (Brandão-Costa et al., 2020). More recently correlation analysis between β3 integrin and EMT markers found that β3 integrin expression correlated positively with the mesenchymal biomarkers vimentin, fibronectin, ZEB1, and ZEB2 in lung adenocarcinoma cells. A knockdown of αvβ3 integrin in lung cancer cells also led cells reverting back to a partial EMT that was independent of TGFβ signaling (Kariya et al., 2021), again suggesting that EMT may be driven by crosstalk between TGFβ and αvβ3 integrin. Finally, knockdown of αvβ3 integrin suppressed TGFβ-induced Notch signaling in human umbilical vein endothelial cells (Wang et al., 2018) which is also involved in the fibrotic process (Condorelli et al., 2021; Zhang et al., 2024).

In conclusion, this study shows that a switch in integrin expression and activity in old TM cells contributes to the development of a mesenchymal phenotype within the TM by upregulating the expression of *αSMA*, *VIM*, *TWIST1*, and *SNAI*2. Ultimately, integrins are likely to play a multi-faceted and essential role in controlling EMT/EndMT in the TM. They regulate cell contractility and adhesion, and are one of the major classes of receptors that respond to mechanoenvironmental cues such as stiffness that are involved in the development of a myofibroblast phenotype and also detected in glaucomatous TM tissues (Last et al., 2011). They also influence numerous downstream signaling pathways involved in EndMT, including TGFβ2 signaling, focal adhesion kinase (FAK)/p21-activated kinase (PAK), Wnt/β-catenin, FAK/glycogen synthase kinase-3β (GSK3β) and ILK/NF-kB (Piera-Velazquez and Jimenez, 2019). Hence, the role of these integrins in the early development of a mesenchymal phenotype makes them potential targets for therapeutic interventions in the development of POAG.

## Data Availability

The original contributions presented in the study are included in the article, further inquiries can be directed to the corresponding author.

## References

[B1] Arjonen A. Alano J. Veltel S. Ivaska J. (2012). Distinct recycling of active and inactive β1 integrins. Traffic 13, 610–625. 10.1111/j.1600-0854.2012.01327.x 22222055 PMC3531618

[B2] Bouvard D. Pouwels J. De Franceschi N. Ivaska J. (2013). Integrin inactivators: balancing cellular functions in vitro and in vivo. Nat. Rev. Mol. Cell Biol. 14, 430–442. 10.1038/nrm3599 23719537

[B3] Brandão-Costa R. M. Helal-Neto E. Vieira A. M. Barcellos-De-Souza P. Morgado-Diaz J. Barja-Fidalgo C. (2020). Extracellular matrix derived from high metastatic human breast cancer triggers epithelial-mesenchymal transition in epithelial breast cancer cells through αvβ3 integrin. Int. J. Mol. Sci. 21, 2995. 10.3390/ijms21082995 32340328 PMC7216035

[B4] Burridge K. Chrzanowska-Wodnicka M. (1996). Focal adhesions, contractility, and signaling. Ann Rev Cell and Dev Biol 12, 463–518. 10.1146/annurev.cellbio.12.1.463 8970735

[B5] Calderwood D. A. Tai V. Di Paolo G. De Camilli P. Ginsberg M. H. (2004). Competition for talin results in trans-dominant inhibition of integrin activation. J. Biol. Chem. 279, 28889–28895. 10.1074/jbc.M402161200 15143061

[B6] Caswell P. T. Vadrevu S. Norman J. C. (2009). Integrins:Masters and slaves of endocytic transport. Nat. Rev. Mol. Cell Biol. 10, 843–853. 10.1038/nrm2799 19904298

[B7] Condorelli A. G. Hachem M. E. Zambruno G. Nystrom A. Candi E. Castiglia D. (2021). Notch-ing up knowledge on molecular mechanisms of skin fibrosis: focus on the multifaceted notch signalling pathway. J. Biomed. Sci. 28, 36. 10.1186/s12929-021-00732-8 33966637 PMC8106838

[B8] Diaz-Gonzalez F. Forsyth J. Steiner B. Ginsberg M. H. (1996). Trans-dominant inhibition of integrin function. Mol. Biol. Cell 7, 1939–1951. 10.1091/mbc.7.12.1939 8970156 PMC276041

[B9] Dupuy A. G. Caron E. (2008). Integrin-dependent phagocytosis: spreading from microadhesion to new concepts. J. Cell Sci. 121, 1773–1783. 10.1242/jcs.018036 18492791

[B10] Ehrlich J. R. Burke-Conte Z. Wittenborn J. S. Saaddine J. Omura J. D. Friedman D. S. (2024). Prevalence of glaucoma among US adults in 2022. JAMA Ophthalmol. 142, 1046–1053. 10.1001/jamaophthalmol.2024.3884 39418040 PMC11581589

[B11] Fang J. S. Hultgren N. W. Hughes C. C. W. (2021). Regulation of partial and reversible endothelial-to-mesenchymal transition in angiogenesis. Front. Cell Dev. Biol. 9, 702021. 10.3389/fcell.2021.702021 34692672 PMC8529039

[B12] Faralli J. A. Gagen D. Filla M. S. Crotti T. N. Peters D. M. (2013). Dexamethasone increases αvβ3 integrin expression and affinity through a calcineurin/NFAT pathway. BBA- Mol. Cell Res. 1833, 3306–3313. 10.1016/j.bbamcr.2013.09.020 24100160 PMC3864090

[B13] Faralli J. A. Filla M. S. Peters D. M. (2019a). Effect of αvβ3 integrin expression and activity on intraocular pressure (IOP). Invest. Ophthalmol. Vis. Sci. 60, 1776–1788. 10.1167/iovs.18-26038 31022732 PMC6485315

[B14] Faralli J. A. Filla M. S. Peters D. M. (2019b). Role of fibronectin in primary open angle glaucoma. Cells 8, 1518. 10.3390/cells8121518 31779192 PMC6953041

[B15] Faralli J. A. Filla M. S. Peters D. M. (2022). Integrin crosstalk and its effect on the biological functions of the trabecular meshwork/Schlemm's canal. Front. Cell Dev. Biol. 10, 886702. 10.3389/fcell.2022.886702 35573686 PMC9099149

[B16] Faralli J. A. Filla M. S. Peters D. M. (2023). Role of integrins in the development of fibrosis in the trabecular meshwork. Front. Ophthalmol. 3, 1274797. 10.3389/fopht.2023.1274797 38983065 PMC11182094

[B17] Filla M. Woods A. Kaufman P. L. Peters D. M. (2006). Β1 and β3 integrins cooperate to induce syndecan-4-containing cross-linked actin networks in human trabecular meshwork cells. Invest. Ophthalmol. Vis. Sci. 47, 1956–1967. 10.1167/iovs.05-0626 16639003 PMC1511964

[B18] Filla M. Schwinn M. K. Sheibani N. Kaufman P. L. Peters D. M. (2009). Regulation of cross-linked actin network (CLAN) formation in human trabecular meshwork (HTM) cells by convergence of distinct β1 and β3 integrin pathways. Invest. Ophthalmol. Vis. Sci. 50, 5723–5731. 10.1167/iovs.08-3215 19643963 PMC3003706

[B19] Filla M. S. Faralli J. A. Peotter J. L. Peters D. M. (2017). The role of integrins in glaucoma. Exp. Eye Res. 158, 124–136. 10.1016/j.exer.2016.05.011 27185161 PMC5612317

[B20] Filla M. Faralli J. A. Desikan H. Peotter J. L. Wannow A. C. Peters D. M. (2019). Activation of αvβ3 integrin alters fibronectin fibril formation in human trabecular meshwork cells in a Rock-independent manner. Invest. Ophthalmol. Vis. Sci. 60, 3897–3913. 10.1167/iovs.19-27171 31529121 PMC6750892

[B21] Filla M. S. Meyers K. A. Faralli J. A. Peters D. M. (2021). Overexpression and activation of αvβ3 integrin differentially affects TGFβ2 signaling in human trabecular meshwork cells. Cells 10, 1923. 10.3390/cells10081923 34440692 PMC8394542

[B22] Filla M. S. Faralli J. A. Dunn C. R. Khan H. Peters D. M. (2023). NFATc1 regulation of dexamethasone-induced TGFβ2 expression is cell cycle dependent in trabecular meshwork cell. Cells 12, 504. 10.3390/cells12030504 36766846 PMC9914240

[B23] Fuchshofer R. Tamm E. R. (2012). The role of TGF−β in the pathogenesis of primary open-angle glaucoma. Cell Tissue Res. 347, 279–290. 10.1007/s00441-011-1274-7 22101332

[B24] Gagen D. Filla M. S. Clark R. Liton P. Peters D. M. (2013). Activated αvβ3 integrin regulates αvβ5 integrin-mediated phagocytosis in trabecular meshwork cells. Invest. Ophthalmol. Vis. Sci. 54, 5000–5011. 10.1167/iovs.13-12084 23821196 PMC3723377

[B25] Galliher A. J. Schiemann W. P. (2006). Β3 integrin and src facilitate transforming growth factor-β mediated induction of epithelial-mesenchymal transition in mammary epithelial cells. Breast Cancer Res. 8, R42. 10.1186/bcr1524 16859511 PMC1779461

[B26] Gingras M.-E. Larouche K. Larouche N. Leclerc S. Salesse C. Guerin S. L. (2003). Regulation of the integrin subunit alpha5 gene promoter by the transcription factors Sp1/Sp3 is influenced by the cell density in rabbit corneal epithelial cells. Invest. Ophthalmol. Vis. Sci. 44, 3742–3755. 10.1167/iovs.03-0191 12939287

[B27] Gingras M.-E. Masson-Gadais B. Zaniolo K. Leclerc S. Drouin R. Germain L. (2009). Differential binding of the transcription factors Sp1, AP-1, and NFI to the promoter of the human alpha5 integrin gene dictates its transcriptional activity. Invest. Ophthalmol. Vis. Sci. 50, 57–67. 10.1167/iovs.08-2059 18775869

[B28] Goffin J. M. Pittet P. Csucs G. Lussi J. W. Meister J.-J. Hinz B. (2006). Focal adhesion size controls tension-dependent recruitment of α-smooth muscle actin to stress fibers. J. Biol. Chem. 172, 259–268. 10.1083/jcb.200506179 16401722 PMC2063555

[B29] Hennig R. Kuespert S. Haunberger A. Goepferich A. Fuchshofer R. (2016). Cyclic RGD peptides target human trabecular meshwork cells while ameliorating connective tissue growth factor-induced fibrosis. J. Drug Target 24, 952–959. 10.3109/1061186X.2016.1163709 26973018

[B30] Hinz B. (2010). The myofibroblast: Paradigm for a mechanically active cell. J. Biomech. 43, 146–155. 10.1016/j.jbiomech.2009.09.020 19800625

[B31] Hinz B. Gabbiani G. (2010). Fibrosis: recent advances in myofibroblast biology and new therapeutic perspectives. F1000 Biol. Rep. 2, 78. 10.3410/B2-78 21170369 PMC2998803

[B32] Hinz B. Phan S. H. Thannickal V. J. Prunotto M. Desmouliere A. Varga J. (2012). Recent developments in myofibroblast biology: paradigms for connective tissue remodeling. Am. J. Path 180, 1340–1355. 10.1016/j.ajpath.2012.02.004 22387320 PMC3640252

[B33] Hinz B. Mcculloch C. A. Coelho N. M. (2019). Mechanical regulation of myofibroblast phenoconversion and collagen contraction. Exp. Cell Res. 379, 119–128. 10.1016/j.yexcr.2019.03.027 30910400

[B34] Islam S. Boström K. I. Di Carlo D. Simmons C. A. Tintut Y. Yao Y. (2021). The mechanobiology of endothelial-to-mesenchymal transition in cardiovascular disease. Front. Physiol. 12, 734215. 10.3389/fphys.2021.734215 34566697 PMC8458763

[B35] Johns K. Faralli J. A. Filla M. S. Shah N. Sun Y. Y. Keller K. E. (2025). Age-related dysregulation of α5β1 and αvβ3 integrin activity alters contractile properties of trabecular meshwork cells. Invest. Ophthalmol. Vis. Sci. 66, 31. 10.1167/iovs.66.6.31 40488712 PMC12161370

[B36] Johnstone M. Xin C. Tan J. Martin E. Wen J. Wang R. K. (2021). Aqueous outflow regulation – 21st century concepts. Prog. Retin Eye Res. 83, 100917. 10.1016/j.preteyeres.2020.100917 33217556 PMC8126645

[B37] Jones C. Ehrlich H. P. (2011). Fibroblast expression of α-smooth muscle actin, α2β1 integrin and αvβ3 integrin: influence of surface rigidity. Exp. Mol. Pathol. 91, 394–399. 10.1016/j.yexmp.2011.04.007 21530503 PMC3139750

[B38] Junglas B. Yu A. H. L. Welge-Lussen U. Tamm E. Fuchshofer R. (2009). Connective tissue growth factor induces extracellular matrix deposition in human trabecular meshwork cells. Exp. Eye Res. 88, 1065–1075. 10.1016/j.exer.2009.01.008 19450452

[B39] Junglas B. Kuespert S. Seleem A. A. Struller T. Ullmann S. Bosl M. (2012). Connective tissue growth factor causes glaucoma by modifying the actin cytoskeleton of the trabecular meshwork. Am. J. Pathol. 180, 2386–2403. 10.1016/j.ajpath.2012.02.030 22542845

[B40] Kariya Y. Oyama M. Suzuki T. Kariya Y. (2021). αvβ3 integrin induces partial emt independent of TGF-β signaling. Comms Biol. 4, 490. 10.1038/s42003-021-02003-6 33883697 PMC8060333

[B41] Kechagia J. Z. Ivaska J. Roca-Cusachs P. (2019). Integrins as biomechanical sensors of the microenvironment. Nat. Rev. Mol. Cell Biol. 20, 457–473. 10.1038/s41580-019-0134-2 31182865

[B42] Keller K. E. Peters D. M. (2022). Pathogenesis of glaucoma: extracellular matrix dysfunction in the trabecular meshwork—A review. Clin. Exp. Ophthalmol. 50, 163–182. 10.1111/ceo.14027 35037377 PMC9199435

[B43] Keller K. E. Bhattacharya S. K. Borrás T. Brunner T. M. Chansangpetch S. Clark A. F. (2018). Consensus recommendations for trabecular meshwork cell isolation, characterization and culture. Exp. Eye Res. 171, 164–173. 10.1016/j.exer.2018.03.001 29526795 PMC6042513

[B44] Kim C. Ye F. Ginsberg M. H. (2011). Regulation of integrin activation. Annu. Rev. Cell Dev. Biol. 27, 321–345. 10.1146/annurev-cellbio-100109-104104 21663444

[B45] Lamouille S. Xu J. Derynck R. (2014). Molecular mechanisms of epithelial–mesenchymal transition. Nat. Rev. Mol. Cell Biol. 15, 178–196. 10.1038/nrm3758 24556840 PMC4240281

[B46] Last J. A. Pan T. Ding Y. Reilly C. M. Keller K. E. Acott T. S. (2011). Elastic modulus determination of normal and glaucomatous human trabecular meshwork. Invest. Ophthamol Vis. Sci. 52, 2147–2152. 10.1167/iovs.10-6342 21220561 PMC3080174

[B47] Li H. Raghunathan V. Stamer W. D. Ganapathy P. S. Herberg S. (2022). Extracellular matrix stiffness and TGFβ2 regulate yap/taz activity in human trabecular meshwork cells. Front. Cell Dev. Biol. 10, 844342. 10.3389/fcell.2022.844342 35300422 PMC8923257

[B48] Lolo F.-N. Pavón D. M. Grande-García A. Elosegui-Artola A. Segatori V. I. Sánchez S. (2022). Caveolae couple mechanical stress to integrin recycling and activation. eLife 11, e82348. 10.7554/eLife.82348 36264062 PMC9747151

[B49] Lovisa S. Fletcher-Sananikone E. Sugimoto H. Hensel J. Lahiri S. Hertig A. (2020). Endothelial-to-mesenchymal transition compromises vascular integrity to induce myc-mediated metabolic reprogramming in kidney fibrosis. Sci. Signal 13, eaaz2597. 10.1126/scisignal.aaz2597 32518142 PMC7790440

[B50] Lu F. Zhu L. Bromberger T. Yang J. Yang Q. Liu J. (2022). Mechanism of integrin activation by talin and its cooperation with kindlin. Nat. Commun. 13, 2362. 10.1038/s41467-022-30117-w 35488005 PMC9054839

[B51] Machado-Costa R. Helal-Neto E. Vieira A. M. Barcellos-De-Souza P. Morgado-Diaz J. Barja-Fidalgo C. (2020). Extracellular matrix derived from high metastatic human breast cancer triggers epithelial-mesenchymal transition in epithelial breast cancer cells through αvβ3 integrin. Int. J. Mol. Sci. 21, 2992. 32340328 10.3390/ijms21082995 PMC7216035

[B52] Madamanchi A. Zijlstra A. Zutter M. M. (2014). Flipping the switch: integrin switching provides metastatic competence. Sci. Signal 7 (318), pe9. 10.1126/scisignal.2005236 24667375 PMC4209128

[B53] Moser M. Legate K. R. Zent R. Fassler R. (2009). The tail of integrins, talin and kindlins. Sci 324, 895–899. 10.1126/science.1163865 19443776

[B54] Parvani J. G. Gujrati M. D. Mack M. A. Schiemann W. P. Lu Z. R. (2015). Silencing β3 integrin by targeted ECO/siRNA nanoparticles inhibits emt and metastasis of triple-negative breast cancer. Cancer Res. 75, 2316–2325. 10.1158/0008-5472.CAN-14-3485 25858145 PMC4452414

[B55] Pattabiraman P. Rao P. (2015). Hic-5 regulates actin cytoskeletal reorganization and expression of fibrogenic markers and myocilin in trabecular meshwork cells. Invest. Ophthamol Vis. Sci. 56, 5656–5669. 10.1167/iovs.15-17204 26313302 PMC4553930

[B56] Peotter J. L. Phillips J. Tong T. Dimeo K. Gonzalez J. M. Jr Peters D. M. (2016). Involvement of Tiam1, Rhog, Elmo/Ilk and Rac1-mediated phagocytosis in human trabecular meshwork cells. Exp. Eye Res. 347, 301–311. 10.1016/j.yexcr.2016.08.009 27539661 PMC5333770

[B57] Picht G. Welge-Lussen U. Grehn F. Lütjen-Drecoll E. (2001). Transforming growth factor β2 levels in the aqueous humor in different types of glaucoma and the relation to filtering bleb development. Graefe’s Arch. Clin. Exp. Ophthalmol. 239, 199–207. 10.1007/s004170000252 11405069

[B58] Piera-Velazquez S. Jimenez S. A. (2019). Endothelial to mesenchymal transition: role in physiology and in the pathogenesis of human diseases. Physiol. Rev. 99, 1281–1324. 10.1152/physrev.00021.2018 30864875 PMC6734087

[B59] Prunotto M. Bruschi M. Gunning P. Gabbani G. Weibel F. Ghiggeri G. M. (2015). Stable incorporation of α-smooth muscle actin into stress fibers is dependent on specific tropomyosin isoforms. Cytoskeleton 72, 257–267. 10.1002/cm.21230 26147585

[B60] Rapisarda V. Borghesan M. Miguela V. Encheva V. Snijders A. P. Lujambio A. (2017). Integrin beta-3 regulates cellular senescence by activating the TGF-β pathway. Cell Rep. 18, 2480–2493. 10.1016/j.celrep.2017.02.012 28273461 PMC5357738

[B61] Roca-Cusachs P. Iskratsch T. Sheetz M. P. (2012). Finding the weakest link - exploring integrin-mediated mechanical molecular pathways. J. Cell Sci. 125, 3025–3038. 10.1242/jcs.095794 22797926 PMC6518164

[B62] Schoenwaelder S. M. Burridge K. (1999). Bidirectional signaling between the cytoskeleton and integrins. Curr. Opin. Cell Biol. 11, 274–286. 10.1016/s0955-0674(99)80037-4 10209151

[B63] Selman M. Pardo A. (2021). Fibroageing: an ageing pathological feature driven by dysregulated extracellular matrix-cell mechanobiology. Ageing Res. Rev. 70, 101393. 10.1016/j.arr.2021.101393 34139337

[B64] Singh P. Carraher C. Schwarzbauer J. E. (2010). Assembly of fibronectin extracellular matrix. Annu. Rev. Cell Dev. Biol. 26, 397–419. 10.1146/annurev-cellbio-100109-104020 20690820 PMC3628685

[B65] Stamer W. D. Acott T. S. (2012). Current understanding of conventional outflow dysfunction in glaucoma. Curr. Opinon Ophthalmol. 23, 135–143. 10.1097/ICU.0b013e32834ff23e 22262082 PMC3770936

[B66] Stamer W. D. Braakman S. T. Zhou E. H. Ethier C. R. Fredberg J. J. Overby D. R. (2015). Biomechanics of schlemm’s canal endothelium and intraocular pressure reduction. Prog. Retin. Eye Res. 44, 86–98. 10.1016/j.preteyeres.2014.08.002 25223880 PMC4268318

[B67] Sun Y. Hamlin A. J. Schwarzbauer J. E. (2025). Fibronectin matrix assembly at a glance. J. Cell Sci. 138, 263834. 10.1242/jcs.263834 40130407 PMC12050093

[B68] Tan J. C. Ko M. K. Woo K. L. Kelber J. A. (2024). Aqueous humor TGFβ and fbrillin-1 in tsk mice reveal clues to poag pathogenesis. Sci. Rep. 14, 3517. 10.1038/s41598-024-53659-z 38347040 PMC10861487

[B69] Tang D. D. Anfinogenova Y. (2009). Physiologic properties and regulation of the actin cytoskeleton in vascular smooth muscle. J. Cardiovasc. Pharmacol Ther 13, 130–140. 10.1177/1074248407313737 18212360 PMC2396785

[B70] Tham Y.-C. Li X. Wong T. Y. Quigley H. A. Aung T. Cheng C.-Y. (2014). Global prevalence of glaucoma and projections of glaucoma burden through 2040: a systematic review and meta-analysis. Ophthalmol 121, 2081–2090. 10.1016/j.ophtha.2014.05.013 24974815

[B71] Tripathi R. C. Li J. Chan W. F. Tripathi B. J. (1994). Aqueous humor in glaucomatous eyes contains an increased level of TGF-β2. Exp. Eye Res. 59, 723–727. 10.1006/exer.1994.1158 7698265

[B72] Truong H. H. Xiong J. Ghotra V. P. S. Nirmala E. Haazen L. Le Devedec S. E. (2014). Β1 integrin inhibition elicits a prometastatic switch through the TGFβ-mir-200-Zeb network in E-cadherin-positive triple-negative breast cancer. Sci. Signal 7, ra15. 10.1126/scisignal.2004751 24518294

[B73] Van Zyl T. Yan W. Mcadams A. Peng Y.-R. Shekhar K. Regev A. (2020). Cell atlas of aqueous humor outflow pathways in eyes of humans and four model species provides insight into glaucoma pathogenesis. Proc. Natl. Acad. Sci. U. S. A. 117, 10339–10349. 10.1073/pnas.2001250117 32341164 PMC7229661

[B74] Wang W. Wang Z. Tian D. Zeng X. Liu Y. Fu Q. (2018). Integrin β3 mediates the endothelial-to-mesenchymal transition via the notch pathway. Cell Physiol. Biochem. 49, 985–997. 10.1159/000493229 30196283

[B75] Yang Y.-F. Holden P. Sun Y. Y. Faralli J. A. Peters D. M. Keller K. E. (2025). Fibrosis-related gene expression in normal and glaucomatous trabecular meshwork cells. Invest. Ophthalmol. Vis. Sci. 66, 48. 10.1167/iovs.66.3.48 40126508 PMC11951066

[B76] Zhang X. Xu Z. Chen Q. Zhou Z. (2024). Notch signaling regulates pulmonary fibrosis. Front. Cell Dev. Biol. 12, 1450038. 10.3389/fcell.2024.1450038 39450276 PMC11499121

[B77] Zhou L. Maruyama I. Li Y. Cheng E. L. Yue B. Y. J. T. (1999). Expression of integrin receptors in the human trabecular meshwork. Curr. Eye Res. 19, 395–402. 10.1076/ceyr.19.5.395.5297 10520215

